# General Analytical Schemes for the Characterization of Pectin-Based Edible Gelled Systems

**DOI:** 10.1100/2012/967407

**Published:** 2012-05-03

**Authors:** Maryam Haghighi, Karamatollah Rezaei

**Affiliations:** Department of Food Science, Engineering and Technology, University of Tehran, Karaj 31587-77871, Iran

## Abstract

Pectin-based gelled systems have gained increasing attention for the design of newly developed food products. For this reason, the characterization of such formulas is a necessity in order to present scientific data and to introduce an appropriate finished product to the industry. Various analytical techniques are available for the evaluation of the systems formulated on the basis of pectin and the designed gel. In this paper, general analytical approaches for the characterization of pectin-based gelled systems were categorized into several subsections including physicochemical analysis, visual observation, textural/rheological measurement, microstructural image characterization, and psychorheological evaluation. Three-dimensional trials to assess correlations among microstructure, texture, and taste were also discussed. Practical examples of advanced objective techniques including experimental setups for small and large deformation rheological measurements and microstructural image analysis were presented in more details.

## 1. Introduction

Pectin, a heteropolysaccharide of plants' cell wall, is a well-known gelling agent for food and drug applications. The power of pectin to revolutionize the structure of a sol system to generate gel network, along with its plant-originated nature and numerous health-beneficial properties, have resulted in its ever-growing applications for creating edible gelled systems. In fact, a combination of several useful characteristics such as being plant-based, possessing functionality, safety at high levels, commercial availability in various adjusted types for different products, low cost, and ease of production and application are some of the advantages of pectins over many other gelling agents [1–4]. Gel character of an edible product can be advantageous in many ways. Typical examples can be as simple as the anticipated pleasure and relaxed feeling of the smooth texture of a gel dessert [5], or on the other hand the valuable technical applications of gels for facilitating intake of bitter drugs [6] or insitu controlled release of drugs in specific pharmaceutical applications [7]. For food industry, pectins have been used in various products including beverages, confectionary, bakery, dairy, and meat products [1–4]. The backbone of pectin molecule is a linear chain of *α*(1,4)-D-galacturonic acid units interrupted occasionally by (1,2)-L-rhamnose residues. Pectins can be divided into two structural groups: high methoxyl pectins (HMPs) with a degree of estrification (DE) (or methoxylation/methylation, DM), of more than 50% and low methoxyl pectins (LMPs) with a DE of less than 50% [1]. Some of the carboxyl groups of galacturonic acid units can be substituted with amide groups. These kinds of pectins are called amidated low methoxyl pectins (ALMPs) and are characterized by the degree of amidation (DA) [1, 3]. The intrinsic factors such as DE and DA [8], degree of polymerization (DP) or chain length, and methoxylation patterns [9] are key parameters affecting the behavior of pectins. Furthermore, extrinsic factors such as pectin and calcium concentrations [9], pH, temperature, total soluble solids (TSSs), and different types of sugars, metal ions, and bulking agents [1, 8] significantly influence the characteristics of a pectin-based system. Several interactions may be anticipated when pectin molecules are used along with other molecules such as different carbohydrates or proteins in the matrix of a formulated product. HMPs, LMPs, and ALMPs have different mechanisms of gelation [3, 10, 11]. For example, complexes of calcium ions and electronegative cavities formed by galacturonic acid residues of two LMP chains result in the formation of junction zones [12]. An appropriate amount of divalent ions (e.g., calcium) and a proper number of successive nonmethoxylated galacturonic residues are needed to form adequate junction zones and consequently a true LMP gel [1, 3, 8, 9, 12]. ALMPs form gel through the both calcium complexes and hydrogen bonds [8]. It has been reported that ALMP shows a higher degree of thermoreversibility [8]. In general, while benefits and efficacy of pectin-based edible systems are well known, insight into these types of systems is very complicated. Numerous studies have been carried out to approach definitions and understandings of pectin-based gelled systems. Therefore, a number of common evaluation methods are used to characterize pectin-based systems. In the present paper, analytical methods for the evaluation of pectin-based systems are divided into five major groups: physicochemical, observational, rheological, microstructural, and psychorheological. Emphasis is made on the most common principles and practices for each group.  Table 1 shows the overview of the common, advanced, and conceptual analytical schemes discussed in this study for the characterization of pectin-based edible gel systems.

## 2. Physicochemical Analysis

Physicochemical analyses are usually the first step because of the fact that these tests define the preliminary conditions of the sample. The most common tests under this category are measurements of total soluble solids and pH. These are important extrinsic factors for jellification [1]. However, investigating the DM and DA of the pectins may be regarded as other first-step tests carried out by soponification and HPLC methods. This is usually performed when an unidentified pectic extract from a new source is used [17]. In addition to that, molecular size distribution or the average molar mass of heteropolysaccharides in such extracts can be measured by size-exclusion chromatography using light scattering detection system [12, 20, 24]. Also, Syneresis and turbidity are usually assessed.

### 2.1. Total Soluble Solids and pH

Gelation of pectin-based systems may be affected by the solutes used in the formula (e.g., sugars) and pH value of the system. When it is necessary to report total soluble solids (TSSs) of the designed pectin-based gel formulations, a refractometer is used and TSS is expressed as °Brix [13, 27, 28]. It is very common to use citrate buffer to prepare pectin solutions. This is used for the studies in which evaluation of other parameters in a constant pH condition is desired [3, 10, 16, 21]. To study the influence of pH on gelation in ALMP-based systems, Lootens et al. [18] took advantage of glucono delta-lactone (GDL), which is capable of decreasing the pH *in situ* [18]. A pH meter is used to record pH values of the samples when using GDL to reduce the pH [8, 18], or when fruit juice or distilled water is used for the preparation of pectin solutions and also in order to let the system live with its natural pH [27].

### 2.2. Turbidity

Gels are often regarded as translucent materials. However, new formulations, incorporation of new ingredients such as functional ingredients, application of mixtures of gelling agents (e.g., gelatin/gellan, HMP/ALMP, etc.), and different preparation methods may result in turbidity. Werner et al. [29] determined the turbidity of gelling and nongelling pectin systems by using a temperature-controlled Helios gamma spectrophotometer (Thermo Spectronic, Cambridge, UK) at 500 nm [29]. The following expression was used to report the turbidity value (*τ*):

(1) $$\tau =\left(\frac{-1}{L}\right)\ln\left(\frac{I_{t}}{I_{0}}\right),$$

where *L* is the light path width in the cell (typically 1 cm), *I*_*t*_ is the transmitted light intensity, and *I*_0_ is the incident light intensity [29]. Measurement of the turbidity of gellan/gelatin mixed gels with spectrophotometric methods has been reported [30], which may also be applicable for pectin-based gels. In the study carried out by Lau et al. [30], hot polymer solution was poured into 1 cm plastic couettes. The samples were let set at appropriate temperature and time. Turbidity was then measured using a Pye Unicam PU 8600 UV/Visible spectrophotometer (Pye Unicam Ltd., Cambridge, UK) at 550 nm against distilled water. Turbidity was linked to the type and concentration of gelling agents, calcium concentration, and, as a result, to the light scattering aggregate-formation upon cooling [30].

### 2.3. Syneresis

Liquid exudation from the gel network is a postgelation phenomenon called syneresis [31]. Thermodynamically, gel is in metastable state. Therefore, no constant textural behavior should be expected as a result of possible structural rearrangements. A number of bonds within the network may reorganize releasing the water or soluble part reserved within the body of gel during gel formation [31]. Syneresis has been related to pH, temperature [1, 23, 31], type and ratio of ingredients incorporated (e.g., application of high methoxyl pectins, low methoxyl pectins or pectins with different degrees of calcium sensitivity), and possible interactions between them [17, 23]. Gel syneresis event may be evaluated immediately after gel network formation, at the end of the first day or in more extended periods, for example with specified intervals in a few months after the production. For the latter, gel aging may be a more suitable descriptive phrase. Methods of gel syneresis evaluation and frequency of such evaluations depend on the type of the sample and the anticipated shelf life. A common method may be placing an inverted gel container (e.g., jelly jar or tube) on a graduated cylinder and monitoring the amount of water separated. When gel is prepared in large bulk, a weighted part of the sample may be placed on a folded filter paper in a funnel located on the top of a graduated cylinder. The percentage of syneresis is calculated from the following equation [28]:

(2) $$\begin{array}{cc} & \text{Syneresis}\;\left(\%\right)\\ & \;\;=\frac{\text{Total}\;\text{weight}\;\text{of}\;\text{separated}\;\text{liquid}\;\left(\text{g}\right)\times \;100}{\text{Total}\;\text{weight}\;\text{of}\;\text{the}\;\text{gel}\;\text{sample}\;\left(\text{g}\right)}. \end{array}$$

Jha et al. [28] repeated the syneresis test on days 15, 30, and 60 after preparation [28]. Withdrawing the separated liquid by a Pasteur pipette from the gel container is a practical approach when the amount of released water is high [23]. When the amount of separated water is low, placing a weighed sample of the gel between filter papers for the determination of weight loss seems to be a suitable method (due to water absorption by papers). This method is similar to serum separation determination methods that are usually applied for meat gels [32]. However, the centrifugation step may be omitted due to the acceptable weaker structure of pectin gels compared to meat gels. In some study designs, distinguishing gel samples showing syneresis (regardless of its severity) was important. Thus, observation of water at the gel surface was considered as syneresis [17]. Similarly, a simple visual judgment was carried out to identify “weeping gels,” by tilting the sample container and detecting separated water [33]. For high quality gels (with appropriate formula), no syneresis is usually observed at the first day of preparation [5]. On the other hand, a syneresis of 7% of sample mass to as high as a severe syneresis has been addressed for pectin-based gels [23].

## 3. Characterizations Based on Visual Observations

Although not always scientifically convincing, visual judgments seem to play an important role in the evaluation of pectin-based gels. An example was given for syneresis assessment in previous section of this study [17, 21]. Madhav and Pushpalatha [33] reported the turbidity of the gels by visually separating gel samples that appeared cloudy. “Ladle test” has been used to indicate sufficient boiling time for gel sample preparation [28]. The rationale of this test is the visual observation of the sample solution's sheeting off from the edge of a spoon due to the proper consistency. This is an indication of proper heating duration [28]. Estimation of gel setting time (gel time or gel point) has been carried out by visual observations according to “tilting tube method” [23, 29]. The gel sample container (e.g., a tube or a beaker) is slanted a few degrees from vertical condition. If the surface of the sample is remained perpendicular to the walls of the container, the gel is considered set. The visually detected gelation is then compared with instrumental evaluations. Similarly, the phase diagram of the pectin-based system is obtained by visual observations [17]. Pectin-calcium samples are categorized as sol or gel after standing for 48 h at 20°C. The gel samples are regarded as the ones that do not flow or deform under their own weight upon tilting the container. Furthermore, the visually observed sol-gel transition state is reported and used in phase diagrams [17].

## 4. Textural Analysis

Rheological measurements can be considered as the most common evaluation techniques for assessing the textural properties of pectin-based system. Advanced instrumental approaches are used to dynamically monitor sol-gel behavior or gel point (e.g., via temperature sweep mode in an oscillatory test) and to compare viscoelastic texture properties. For the latter, usually, large deformation rheological measurements [34] and small deformation assessments [34, 35] in frequency sweep mode are preferred. Endress et al. [36] presented an overview of the common rheological methods and devices to evaluate pectin-based systems (as gels or solutions). Testing devices such as the ridgelimeter, Herbstreith pectinometer, penetrometers, texture analysers, and advanced rheometers were discussed [36]. Large and small rheological measurements are presented here in more details.

### 4.1. Large Deformation Rheological Measurements

Food materials possess diverse and different rheological properties. These properties greatly affect the characteristics of the final system. Therefore, measurement and interpretation of the rheological properties by the application of appropriate tests are crucial. When the relative magnitude of the imposed deformation is large, the test is called large deformation rheological measurement [34]. Texture profile analysis (TPA) is probably the most common method to evaluate large deformations of food materials. The method was inspired by the action of two bites, imitated with a double-compression mechanical test. Compression is achieved using parallel plates when one plate is fixed and the other plate moves toward the sample, compresses the sample to the desired percentage of original sample height, returns to its original place, and repeats the same procedure. The compression depth indicated as a percentage of original sample height can be different such as 30% [37, 38], 50% [39], 80% [40], and 90% [41]. According to Steffe [40], hardness, cohesiveness, adhesiveness, springiness, and gumminess are common parameters considered for the textural analysis of gels. Hardness is force at maximum compression during the first bite [40, 41]. Hardness could be described by the terms soft, firm, and hard. Measured variable for hardness is force (*mlt* ^−2^) (*m*, mass; *l*, length; *t*, time). Cohesiveness is the ratio of the positive force areas under the first and second compression steps [5, 41]. So, the measured variable has no dimension. Adhesiveness can be described as the negative force area of the first bite representing the work necessary to pull the plunger away from the sample [40]. The measured variable is work (ml^2^t^−2^). Springiness (elastic recovery or elasticity) is defined as the distance the sample recovers after the first compression or the distance from the end of the first bite to the start of the second bite. In most references the measured variable is distance (*l)* [5, 40, 41]. However, it can also be reported as a percentage of the distance related to the sample's maximum compression during the second bite divided by the initial sample height [30]. Also, it has been reported as the ratio of the distance of the maximum compression during the second bite divided by the distance of the maximum compression during the first bite [42]. Gumminess is the product of hardness multiplied by cohesiveness that represents the energy necessary to disintegrate a semisolid food (e.g., gels), making it ready for swallowing, similar to chewiness for solid foods [40]. The experimental setups for large deformation rheological characterizations of some pectin-based systems were listed in Table 2. In addition, the nature of the pectin-based gel samples and reported textural features were presented (Table 2).

### 4.2. Small Deformation Rheological Measurements

Small deformation dynamic rheological measurements (also known as harmonic or oscillatory tests) are conducted by the application of small oscillating stress or strain and recording the responses of the material [34]. The test has received considerable attention as a modern and advanced method for continuous monitoring of the gel behaviors. By offering the possibility of investigating the textural properties (based on the chemical and physical structure) of the specimen at different stages of sample's life (as a sol, pre-gel, gel, or aged gel), the method may be regarded as the most favored rheological test for many scientists interested in research on pectin-gelled systems. In oscillatory tests, the material is subjected to deformation (in rate-controlled instruments) or stress (in stress-controlled instruments) changing harmonically with time [40]. The test can be operated in tension, bulk compression, or shear mode. The latter is the most common mode for food testing. Parallel plate [9], cone and plate [22], or concentric cylinder fixtures (cup and bob or couette) [21] are preferred geometries for gel materials to be subjected to an oscillating strain [43]. Cup and bob may be used for the systems with low viscosity. Couette geometry may also be applied for pectin-based gel systems and it is known for the more stable results during long-time measurements [18]. However, the most common geometry seems to be the cone and plate. It can be more precise and can make a rapid setting of the temperature possible [18, 43]. For the evaluation of gels and in order to avoid destructive deformation, harmonic tests are commonly carried out by small amplitude oscillatory shear techniques (SAOS) [35]. The application of small strain (or stress) is to ensure that the material will behave in a linear viscoelastic manner [10, 35]. To determine the linear viscoelastic region (LVR), usually a strain-sweep test is performed by changing the amplitude of the input signal (sinusoidal strain), while the frequency is maintained constant [9, 25]. In addition to that, strain sweep test may be used to assess strength of the gel [43]. Common performance modes of oscillatory evaluation of gelled systems are as follows: frequency sweep mode at a constant strain and temperature, temperature sweep mode at a constant strain, and frequency, time sweep mode at constant strain, constant frequency and constant (or at a controlled varying) temperature [35, 44]. Time sweep test is very useful in studying the chemorheology (studying the time-dependent textural behavior caused by chemical reactions) of gel materials [43]. Frequency sweep test could be applied in “finger printing” of different food products (such as pectin-based gelled desserts or yogurts) and studying the impacts of formulation and process parameters on the viscoelasticity. Data may be plotted using frequencies given in different preferred units (1 Hz = 1 cycle/s = 2*π* rad/s) [43]. In general, the textural parameters that can be recorded by oscillatory assessments should be mentioned as storage or elastic modulus, *G*′, showing the solid-like properties of the material; loss or viscous modulus, *G*′′, showing the liquid-like or viscous characteristics of the material; phase angle, *δ*, (also known as phase lag, phase shift or mechanical loss angle); tangent of the phase angle (also known as tan (*δ*), loss tangent, or damping factor), which is calculated from the ratio of *G*′′ to *G*′ and varies from zero to infinity representing the tendency of the material to liquid or solid-like behavior; complex modulus, $G^{*}=\sqrt{{\left(G^{\prime }\right)}^{2}+{\left(G^{\prime \prime }\right)}^{2}}$; complex dynamic viscosity, η* = (G*/*ω*) and also the less common parameters of dynamic viscosity, *η*′, out of phase component of the complex viscosity, *η*′′, complex compliance, J*, storage compliance, *J*′, and loss compliance, *J*′′ [35, 43, 44]. Shapes of the graphs obtained from frequency sweep tests could be used to define the systems as dilute solutions, concentrated solutions, or gels [43]. Gels can be classified as true gels, weak gels or strong gels [44]. This kind of distinction is achievable by comparing the magnitudes of *G*′ and *G*′′ and evaluating their frequency dependence [44]. Some of the other definitions or descriptions used for pectin-based systems subjected to rheological measurements are as follows: self-supporting gel (similar to true gel) [23], fragile gel, cured gel [17], pregel, microgel, gel-like structure, weak cross-linked network structure [21], unaged gel [10], and type I to IV gels as defined by Capel et al. [20].

Structure development rate (SDR) of a biopolymer gel is defined as d*G*′/dt and can be determined under either isothermal or nonisothermal conditions [44]. Sol-gel transition, gel point, gel time, and gel temperature are commonly used to indicate the critical points at which the phase changes occur in a solution system with gelation potential [8, 10, 16, 18, 20, 44]. Recording specific extrinsic and intrinsic parameters of gelation (e.g., DE, DA, pH, TSS, etc.) at this critical point would be of significant value to characterize the designed system. However, the gel point is not always easily detected [12, 35]. Choice of method and experimental setup based on the type of the system may be important. A number of methods including the crossover method (i.e., cross-over of *G*′ and *G*′′) and the Winter-Chambon method [35] are available to identify gel point.  Table 3 presents typical examples of experimental set up details applied for the oscillatory tests on pectin-based gel systems.

## 5. Microstructural Image Analysis

Microstructural image analysis can be applied to observe the microscopic structure of pectin-based gels. The method can provide information about the homogeneousness, formation of clusters or aggregates, structural type of mixed gels, particle size characteristics, and arrangements and interactions. In general, there are different methods to obtain microstructural images of food materials that can also be used for the gels. Scanning electron microscopy (SEM) [45], transmission electron microscopy (TEM) [21], cryoscanning electron microscopy (CSEM) [46], atomic flame microscopy (AFM) [47], environmental scanning electron microscopy (ESEM) [46], and confocal laser scanning microscopy (CLSM) [2] are among the techniques applicable for image analysis. In order to get proper images from the gel samples, several gentle preparation steps such as fixation, dehydration and coating of the sample have to be considered. Improper handling the samples during the above stages can result in false interpretation of the real microstructure of the gel. Considering such points, ESEM and CSLM may be more appropriate methods with less sample preparation complexities for special samples [46, 47]. In the study carried out by Arltoft et al. [2], direct immune-staining technique for localizing pectin was used [2]. The method applied a primary antibody conjugated with a fluorophore and was useful to characterize the native microstructure of the gel samples via omitting the washing, fixation, drying, or slicing procedures. The anti-pectin monoclonal antibody JIM5 may be applicable due to its stability and specificity in the internal conditions of the specimen. Using monoclonal antibody probes can be regarded as an appropriate way of localizing one ingredient in relation to others (e.g., pectin and carrageenan in mixed gels) [2]. Examples of applied microstructural image analysis methods for different types of pectin-based gels and the experiment conditions are given in Table 4.

## 6. Psychorheological Evaluations

**Figure 1:**
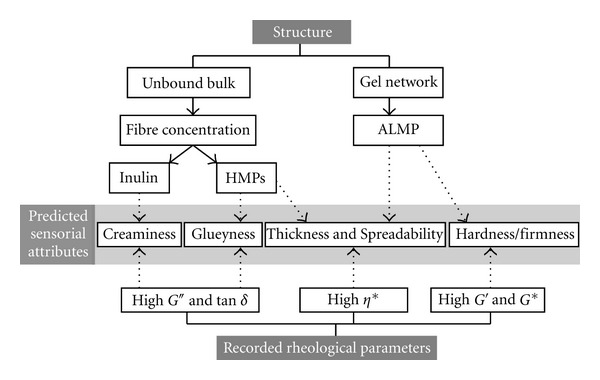
Conceptual model on the relations among structure, rheological properties, and predicted sensory attributes of pectin-based (mixed fibre) functional gel systems formulated by Haghighi et al. [26] (HMPs: high methoxyl pectins; ALMP: amidated low methoxyl pectin; *G*′: storage modulus; *G*′′: loss modulus; η*: complex viscosity; tan *δ*: loss tangent; G*: complex modulus).

Despite the fact that a wide spectrum of useful data can be obtained through the use of the above-mentioned methods, in practice, the final approval of a product is highly dependent on what is perceived by the consumers. Organoleptic/sensory evaluations are designed to evaluate edible products. Nevertheless, even if both objective and subjective tests are satisfactory, they are not really applicable when they are not correlated with each other. Currently, psychorheological evaluations are receiving considerable attention as this kind of study may act as a bridge between the food scientists and the consumers. According to Bourne [5], two types of definitions may be given to psychorheology: scientific- and people-centered. From the scientific point of view, “psychorheology is a branch of psychophysics dealing with the sensory perception of rheological properties of foods.” The second type of the definition may be stated as follows: “psychorheology is the relationship between the consumer preferences and rheological properties of the foods” [5]. A more comprehensive approach would be to study the correlations of microstructural, textural, and sensorial analysis and designing models based on this three-dimensional trail. Relating the structure and texture to organoleptic properties may be regarded as the most advanced and beneficial methods to evaluate the finished products such as pectin-based gels. In general, all procedures to design and process food products deal with a common goal of gaining consumers satisfaction. Therefore, attempts have been made to correlate instrumental measurements to what perceived by the consumers. As mentioned before, TPA tests are amongst the best examples of a mechanical program designed to imitate human bites. Electronic nose and tongue [48] are two new approaches for the instrumental perception of subjective consumer responses. Monge et al. [48] reported the ability of electronic noses to detect gel formation state and the results correlated well with the rheological evaluations in the pectin gel systems [48]. Furthermore, recent attempts have approached a link between the oscillatory measurements and specific sensory properties of pectin gels including sweetness, thickness, and glueyness evaluated by a trained analytical panel [3]. Multivariate statistical approaches can be used to evaluate and correlate the instrumental and sensory data. Amongst the techniques available for examining the relationships in the data sets and to correlate sensory properties and objective measurements, principal component analysis (PCA), and partial least squares (PLSs) analysis are probably the most common methods [49]. PLS Loading plots have been published in the work of Holm et al. [3] for the sensory properties versus pectin types and sensory properties versus rheological parameters [3]. PCA has been used to investigate correlation between microstructural, rheological, and sensory parameters of pectin-incorporated dairy gelled desserts [2] and to study correlation between rheological measurements and the data obtained from the electronic noses in pectin gels [48]. Relations between the microstructure of coarse-stranded pectin gels, syneresis and the so-called “watery” perception or rheological measurements and the “crumbly” perception were discussed in an effort to link taste, texture, and structure of gelled systems [50]. Designing three-dimensional trials (3DTs) for the evaluation and correlation of (micro)structure, texture and taste can lead to insights into true characteristics of formulated gel product. 3DTs or building conceptual models based on the triangular relations of microstructural, rheological, and sensory analysis may result in the ease of predictions and optimizations of different properties of the system. A model representing interrelations between physical and sensorial characteristics of cold set whey protein-polysaccharide composite gels was discussed by van Vliet et al. [50]. The 3DT conceptual model presented in Figure 1 was designed based on the rheological responses of pectin-based functional gels formulated by Haghighi et al. [26]. According to this model, some of the anticipated sensorial attributes were predicted. Renard et al. [31] have reviewed the current status of the research works and information on the relationships of structure, texture and perception of food gels and regarded it as a gap, concluding that although there are scientific principles available in this field, this is not always enough to achieve convinced and desired sensations [31]. Therefore, any efforts to combine various knowledge areas to design new methods capable of reducing this gap would be a new hope.

## 7. Conclusions and Future Trends

Physicochemical analysis, visual observations, textural (rheological) analysis, microstructural analysis, and psychorheological studies are suggested for the evaluation of a new formulated pectin-based edible gel system and also to design perfect assessment procedures. For the physicochemical analysis, simple determinations of total soluble solids and pH are often necessary due to the considerable effects that these parameters can have on the behavior of the final system. Properties such as turbidity, syneresis, and gel point may be rapidly judged by means of visual observations. However, usually more precise methods are required to avoid the subjective misjudgments. Textural measurements can be regarded as the most important stage of the characterization procedures of gelled systems. Texture profile analysis is commonly used and may correlate well with the sensorial results. Dynamic behaviors of the gels such as the changes during the sol-gel transition can be monitored via oscillatory tests. These kinds of tests that are also applicable in different modes of operations are probably the most applied analytical methods in the recent literature on the subject of pectin-based gels. Microstructural image analysis helps the researchers define the microscopic nature of a gel product. Psychorheology is a useful tool to correlate between the sensorial and the rheological data. Furthermore, application of the combined physicochemical-textural analysis or rheological-microstructural techniques (e.g., using a rheomicroscope for monitoring the microstructure of a gel system during the structure formation while recording the related rheological data) may be suggested to improve the efficacy of the evaluations. Three-dimensional trails to correlate structural, textural, and sensorial results may be useful for building models in order to predict and/or optimize the final characteristics of the products. For the future developments, an improved version of 3DTs, as multi-dimensional trails (MDTs) can be suggested that would be a combination of several analytical schemes to evaluate pectin-based systems. It can be of great interest to design novel analytical instruments for MDTs that are capable of recording a wide range of data including physicochemical, textural, microstructural, and sensorial properties of pectin-based gels, simultaneously. Upon gaining satisfactory information about the pectin-based product via the above-mentioned analytical schemes, it would be valuable to perform *in vitro* and *in vivo* assessments in order to learn the biological, nutritional, or functional properties of the consumed product and its final impacts on the consumers.

## Figures and Tables

**Table 1:** An overview of the common, advanced, and conceptual analytical schemes discussed in this study for the characterization of pectin-based edible gel systems.

Common analytical schemes	Common measured parameters
Physicochemical	Total soluble solids, pH, turbidity, syneresis

Observational	Turbidity, syneresis, consistency as an indicator of proper heating duration for gel preparation, gel time or gel point, and so forth

Textural (rheological)	Large deformation (texture profile) analysis for the evaluation of hardness, cohesiveness, adhesiveness, springiness and gumminess, and so forth Small deformation (oscillatory) tests for the evaluation of storage modulus, loss modulus, phase angle, loss tangent, complex modulus, complex dynamic viscosity, gel point or gel time and temperature, and so forth

Microstructural	Homogeneousness, formation of clusters or aggregates, structural type of mixed gels, particle size characteristics, arrangements and interactions, and so forth

Psychorheological	Relationships between sensory properties such as thickness, sweetness, creaminess, and rheological parameters

Advanced and conceptual schemes	Three-dimensional trails (3DTs) including the evaluation and correlation of (micro)structure, texture and taste based on the triangular relations of microstructural, rheological, and sensory analyses
	Multidimensional trails (MDTs), which include the improved version of 3DTs that would be a combination of several analytical schemes to evaluate various parameters of pectin-based systems

**Table 2:** Typical examples of experimental setup and recorded parameters based on large deformation rheological measurements for pectin-based gels.

Sample ingredients	Experimental setup	Recorded parameters	Reference(s)
Strawberry, peach, and pectin (jam)	Puncture test, Stevens LFRA texture analyzer; probe; spherical; 12.7 mm diameter; speed: 0.5 mm/s; penetration depth: 20 mm; samples shape and size: cylindrical, 56 mm diameter	Break load value (gel strength)	Carbonell et al. [13]

ALMP and grape juice (jelly)	TPA, TAX-T2 texturometer (stable microsystems); probe: cylindrical; 2.5 cm diameter; depth: 4 mm; speed: 2 mm/s; sample shape and size: cylindrical, 30 mm diameter; 40 mm height	Hardness Cohesiveness	Sousa et al. [14]

Apple pomace, quince (natural pectin sources), and sugar (jelly)	TPA universal testing machine (MTS synergy 200H) compression depth: 3 mm; speed: 20 mm/min; sample shape and size: rectangular, 1 cm width; 2 cm length, 1 cm thickness	Hardness Cohesiveness	Royer et al. [15]

Fish paste and ALMP (fish gel)	TPA, TA-XT2i texturometer (Stable Micro Systems, Viena Court, England); probe: aluminum cylindrical, P/50, 50 mm diameter, speed: 60 mm/min compression depth: at 75% initial sample height; sample shape and size: cylindrical, 19 mm diameter, 30 mm length	Fracturability Hardness Cohesiveness Springiness	Ramírez et al. [4]

Fish paste and ALMP (fish gel)	Puncture test; probe: spherical, P/0.5 s, 12 mm diameter; speed: 60 mm/min compression depth: 75% initial sample height	Breaking force Gel strength Gel deformation	Ramírez et al. [4]

ALMP and carrageenan, (dairy gelled dessert)	Penetration test, texture analyser TA-XT2i (stable microsystems, Surrey, UK); probe: 1/2 inch cylindrical, Depth: 12 mm, speed: 1 mm/s	Firmness Adhesiveness Fracture point	Arltoft et al. [2]

ALMP: amidated low methoxyl pectin; TPA: texture profile analysis.

**Table 3:** Typical examples of experimental set up and recorded parameters based on small deformation rheological measurements (oscillatory tests) for pectin-based gels.

Sample ingredients	Experimental setup	Recorded parameters	References
HMP and fructose	Carri-med rheometer (Valley View, OH); geometry: cone and plate (4 cm diameter; 2° cone angle); strain: 3%; frequency: 1 Hz; duration: 2 h; temperature sweep: 50–10°C frequency sweep: 0.01–1 Hz; temperatures: 50, 40, 30, 20, 15, and 10°C	*G*′ *G*′′ η* tan *δ* SDR	Rao and Cooley [10]

ALMP, Sucrose, and grape juice	Stress-controlled rheometer (Bohlin CS, ref. GTM-CS-V1); geometry: cylindrical concentric with a double gap (30 mL capacity); strain: 2%; frequency sweep: 0.005–2 Hz, temperatures: 65, 50, 35, 20, and 10°C; speed: 0.5°C/min	*G*′ *G*′′ tan *δ*	Sousa et al. [14]

Pectin, sucrose, and calcium	Stress-controlled rheometer CSL-100 (TA Instruments, Surrey, UK); geometry: cone and plate (4 cm diameter; 1° cone angle; 55 *μ*m truncation); strain: 1.5%; frequency: 0.1–10 Hz, Temperature sweep: 90–20°C; speed: 3°C/min, frequency: 1 Hz	*G*′ *G*′′ η* tan *δ*	Norziah et al. [16]

LMP (olive pectic extract) and CaCl_2_	CVO HR 120 rheometer (Bohlin Instruments); geometry: cone and plate (40 mm diameter; 4° cone angle; 150 *μ*m gap); strain: 1%; frequency:1 Hz; Time sweep: 20 h; temperature: 20°C; Frequency sweep: 0.005–5 Hz	*G*′ *G*′′	Cardoso et al. [17]

LMP, ALMP, CaCl_2_, and GDL	Strain-controlled rheometer (ARES, Rheometric Scientific); geometry: cone and plane geometry (50 mm diameter; 2.3° cone angle) or a couette geometry (32 mm inner radius; 34 mm outer radius); atress-controlled rheometer (AR1000, TA Instruments); geometry: cone and plane (40 mm diameter; 1° cone angle); frequency: 1 Hz; temperature sweep: 80–20 or 5°C; speed: 30°C/min	*G*′ *G*′′ Tg	Lootens et al. [18]

ALMP, Sodium caseinate solution, CaCl_2_·2H_2_O and GDL	Bohlin rheometer (CS50, CVO or CVO-R); stress-controlled geometry: concentric cylinder C25 measurement cell; strain: 0.5%; frequency: 1 Hz; duration: 8 h; temperature: 25°C	*G*′ *G*′′	Matia-Merino et al. [8]

HMP, sorbitol, fructose, glucose, sucrose, xylitol, glycerol or ethane-1,2-diol and trisodium citrate	A sensitive prototype rheometer designed and constructed by R. K. Richardson (Cranfield University, UK); geometry: highly truncated cone and plate (50 mm diameter; 0.05 rad cone angle; 1 mm gap); strain: 0.5%; frequency: 1 rad/s; temperature sweep: 95–5, 5–90°C, and 90–5°C; speed: 1°C/min; frequency sweep: 0.1–100 Hz	*G*′ *G*′′	Tsoga et al. [19]

LMP, ALMP, CaCl_2_, and GDL	Strain-controlled rheometer (ARES, Rheometric Scientific); geometry: plane-plane (50 mm diameter; 1 mm gap) or a stress-controlled rheometer (AR1000, TA Instruments); geometry: cone-plane (60 mm diameter; 1° cone angle); frequency: 1 Hz; temperature sweep: 80–5°C	*G*′ *G*′′ Tg	Capel et al. [20]

HMP, LMP, CaCl_2_·2H_2_O, citrate buffer (pH: 3.5), and different sucrose concentrations	Stresstech rheometer (Reologica Instruments, Lund, Sweden); strain-controlled; geometry: cup and bob; strain: 0.002 frequency: 1 Hz; temperature: 20°C, time sweep: 3 and 16 h frequency sweep (16 h after gel preparation): 0.01–10 Hz	*G*′ *G*′′	Löfgren and Hermansson [21]

ALMP and Carrageenan	Stresstech rheometer (Reologica AB, Lund, Sweden); geometry: plate-serrated plate (25 mm diameter); stress sweep from 0.01 until critical stress at a frequency of 1 Hz	*G*′ *G*′′ η* tan *δ*	Arltoft et al. [2]

LMP (from “nopal” cactus pads) and CaCl_2_	Rheometrics (fluids spectrometer RFS II, piscattaway, NJ, USA); geometry: cone and plate (0.5 cm diameter; 0.04 rad cone angle); strain: 5%; frequency sweep: 1–21.5 rad/s temperature sweeps: 85–5 and 60–5°C; Speed: 1°C/min	*G*′ *G*′′ η* tan *δ*	Cárdenas et al. [22]

HMP, CaCl_2_, water, and enzyme solution	CarriMed CSL-100 rheometer, geometry: cone and plate (6 cm diameter; 2° cone angle), Strain: 1%; frequency: 1 Hz; duration: 160 min; temperature sweep: 30–70°C; speed: 1°C/min	*G*′ *G*′′ η*	O'Brien et al. [23]

Pectin (from buttercup squash fruit) and sugar	Physica UDS 200 rheometer; geometry: cup and bob (17 mL total volume); strain: 1%; frequency sweep: 0.01–10 Hz; temperature sweeps: 90–20, 20–90°C; speed: 1°C/min; frequency: 1 Hz	*G*′ *G*′′	O'Donoghue and Somerfield [24]

Mucin and pectin	Paar Physica MCR 301 rheometer (Anton paar GmbH, Austria); geometry: parallel plate (0.05 mm gap; CP50-1) or a concentric cylinder (DG26.7); measuring system; frequency sweep: 100–0.1 Hz; temperature: 37°C	*G*′ *G*′′ tan *δ*	Sriamornsak and Wattanakorn [25]

Pectins with different DM and calcium	Rotational rheometer (ARES, TA Instruments, USA); strain-controlled geometry: parallel plates (50 mm diameter; 1 mm gap); frequency: 10 rad/s; time sweep: 8 h; frequency sweep: 100–0.1 rad/s; temperature: 25°C	*G*′ *G*′′	Fraeye et al. [9]

LMP and calcium	Rotational controlled stress rheometer AR 2000 (TA Instruments); geometry: cone-plate (2 cm diameter; 4° cone angle; 53 *μ*m gap); time sweep: 8 h, frequency: 1 rad/s; strain: 3%; Frequency sweep: 0.01–100 rad/s	*G*′ *G*′′	Gigli et al. [12]

LMP, HMP, sugar, CaCl_2_·2H_2_O, citrate buffer (pH: 3.5)	Stresstech rheometer (Reologica Instruments, Sweden); strain-controlled; geometry: Cup and bob (volume: 25 mL); sample volume: 15.9 mL; strain: 0.002; frequency: 1 Hz; time sweep: every 20 s for 500–600 sweeps	*G*′ *G*′′ tan *δ*	Holm et al. [3]

ALMP, HMP, inulin, Sorbitol, and CaCl_2_·2H_2_O	Paar Physica MCR 300 rheometer (Anton Paar GmbH, Austria); geometry: parallel plates (50 mm diameter; 1 mm gap); and cone and plate (50 mm diameter; 2° cone angle; 0.05 mm gap); strain: 1%; frequency sweep: 0.1–100 Hz; temperature sweep: 90–5°C; speed: 2°C/min	*G*′ *G*′′ G* η* tan *δ*	Haghighi et al. [26]

HMP: high methoxyl pectin; LMP: low methoxyl pectin; ALMP: amidated low methoxyl pectin; GDL: glucono-delta-lactone; DM: degree of methoxylation; *G*′: storage modulus; *G*′′: loss modulus; η*: complex viscosity; tan *δ*: loss tangent; G*: complex modulus; SDR: structure development rate; Tg: gel temperature.

**Table 4:** Typical examples of microstructural image analysis for pectin-based gels.

Sample ingredients	Experimental conditions	Microstructural interpretations	References
ALMP and acid-induced sodium caseinate gel	CLSM: Leica TCS confocal laser scanning microscope, Fluorescence mode with a 100× 1.3 N.A. oil-immersion objective, with an argon/krypton laser, Application of rhodamine B to identify protein, adding droplets of dye solution into caseinate+pectin solution, adding GDL and stirring	Homogeneousness of the sample, pore sizes, the prevention of the formation of strands and clusters in presence of pectin, an increase in the staining intensity of the protein strands attached to the network without pectin, and so on	Matia-Merino et al. [8]

Mixed HM/LM pectin gel	TEM: Transmission electron microscope (LEO 906E Electron Microscopy Ltd., Cambridge, England), sample size: 1 × 1×1 mm cubes, sample preparation 20 h after gel preparation, Fixation for 20 h in aldehyde solution based on citrate buffer, 2% glutaraldehyde, and 0.1% ruthenium red, dehydration, polymerization, thin sectioning	Microgels, inhomogeneous structure, LMP-Ca clusters surrounded by a coherent gel network of HMP, dense LMP-rich areas, sparse HMP-rich areas, aggregations, and so on	Löfgren and Hermansson [21]

Mixed ALMP and carrageenan gel	CLSM: Leica TSP2 CLSM (Leica, Mannheim, Germany) with an argon/krypton and a helium/neon laser, fitted with a HCX PL APO 40 × numerical aperture 1.2 oil immersion objective, sample size: 20 ×12 × 4 mm, staining the pectin with the antibody, localization of pectin by making cytofluorogram or by the multivariate image feature extraction method	Heterogeneous structure, distinguishing three types of mixed gels resulted from multi-gelling agent formulation: interpenetrating, coupled and phase- separated networks, and so on	Arltoft et al. [2]

HMP: high methoxyl pectin; LMP: low methoxyl pectin; ALMP: amidated low methoxyl pectin; CLSM: Confocal Laser Scanning Microscopy; TEM: Transmission Electron microscopy.
